# VIEshunt: towards a ventricular intelligent and electromechanical shunt for hydrocephalus therapy

**DOI:** 10.1186/s12987-025-00629-w

**Published:** 2025-03-14

**Authors:** Fabian Flürenbrock, Leonie Korn, Dominik Schulte, Anthony Podgoršak, Joris Chomarat, Janina Hug, Tiago Hungerland, Caroline Holzer, David Iselin, Luca Krebs, Rosina Weiss, Markus F. Oertel, Lennart Stieglitz, Miriam Weisskopf, Mirko Meboldt, Melanie N. Zeilinger, Marianne Schmid Daners

**Affiliations:** 1 Institute for Dynamic Systems and Control, ETH Zurich, Zurich, Switzerland; 2 Product Development Group Zurich, ETH Zurich, Zurich , Switzerland; 3 Department of Neurosurgery, University Hospital Zurich, Zurich , Switzerland; 4 Center for Preclinical Development, University Hospital Zurich and University of Zurich, Zurich, Switzerland

**Keywords:** Hydrocephalus, Smart shunt, Intracranial pressure, Cerebrospinal fluid, Patient posture, Pressure and drainage control

## Abstract

**Background:**

Shunt systems for hydrocephalus therapy are commonly based on passive mechanical pressure valves that are driven by the intracranial, intra-abdominal, and hydrostatic pressure. The differential pressure acting on the valve determines the drainage rate of cerebrospinal fluid (CSF) but is not a gauge of the physiological condition of the patient. Internal and external influences can cause over- or underdrainage and lead to pathological levels of intracranial pressure (ICP).

**Methods:**

The first prototype of a ventricular intelligent and electromechanical shunt (VIEshunt) is developed, tested, and compared with previous efforts towards the development of a smart shunt. Its key components are a micro pump, a flow meter, a pressure sensor, an inertial measurement unit, a wireless communication interface, and a microcontroller. The VIEshunt prototype was tested in vitro using a hardware-in-the-loop (HiL) test bench that runs real-time patient simulations involving changes in intracranial and intra-abdominal pressure, as well as changes in posture ranging between supine and upright position. The prototype was subsequently tested in an in vivo pilot study based on an acute ovine animal model (n=1) with infusions of artificial CSF.

**Results:**

During 24 h in vitro testing, the prototype detected the simulated posture changes of the patient and automatically adapted the controller reference. The posture-specific ICP references of 12 mmHg for supine and —3 mmHg for upright position were tracked without offset, thus preventing adverse over- and underdrainage during the investigated HiL test scenario. During acute in vivo testing, the prototype first regulated the mean ICP of a sheep from 22 mmHg down to 20 mmHg. Each of the three subsequent intraventricular bolus infusions of 1 mL saline solution increased mean ICP by approximately 11 mmHg. While natural absorption alone decreased ICP by only 5 mmHg within 9 min, the prototype was able to regulate ICP back to the pre-bolus pressure value within 5 min.

**Conclusion:**

The developed VIEshunt prototype is capable of posture-dependent ICP regulation and CSF drainage control. Smart shunt systems based on VIEshunt could improve patient monitoring and enable optimal physiologic therapy by adapting to the individual patient. To derive statistically relevant conclusions for the performance of VIEshunt, future work will focus on the development of a next generation prototype for use in pre-clinical studies.

## Introduction

Since their clinical introduction more than 70 years ago, shunt systems that drain excessive cerebrospinal fluid (CSF) out of the cranial ventricular system have become the gold standard for hydrocephalus therapy [1, 2]. The fundamental components of a typical shunt system include a proximal catheter that collects excessive CSF from the craniospinal system, a shunt valve that regulates the CSF drainage rate, and a distal catheter that diverts the CSF to another part of the body. A variety of shunt valves with differing levels of complexity exist, ranging from relatively simple ball-in-cone valves to more sophisticated programmable spring-ball-in-cone valves, or diaphragm and slit valves. Despite the significant design differences, all these shunt valves are essentially passive mechanical pressure valves that regulate CSF drainage based on the differential pressure acting on them [3, 4]. Although shunt systems have significantly improved the lives of countless patients, there remain fundamental limitations inherent to their design that render contemporary shunt therapy suboptimal. Even for today’s shunts, achieving a physiologically optimal CSF drainage rate to prevent overdrainage or underdrainage of CSF continues to be a key challenge [5–7]. Draining an excessive amount of CSF can result in ventricular collapse and intracranial hemorrhage. Conversely, draining an insufficient amount of CSF can lead to ineffective shunt therapy and persistently elevated intracranial pressure (ICP). Most commonly, ventriculoperitoneal shunts in which the CSF drainage rate is driven by the ICP, the intra-abdominal pressure (IAP) and the hydrostatic pressure difference are used in clinical practice. This limits the physiological drainage of CSF because ICP and IAP are not directly physiologically related and external influences, such as changes in patient posture, can severely alter the physiological pressures and the hydrostatic pressure difference [8–11]. Several types of anti-siphon devices (ASD) have been developed to counteract the effect of posture-induced overdrainage of CSF, however, until today no ideal ASD exists that is capable of fully preventing this problem [12–14]. Additional common and serious complications associated with hydrocephalus shunt therapy, aside from overdrainage, include infection, obstruction, and dislocation of the shunt catheters [15–20]. All of these complications can ultimately result in shunt failure, necessitating costly and potentially risky surgical shunt revisions for patients. The failure rate of shunts implanted in pediatric hydrocephalus patients have been reported to be approximately 50% after two years [21], while a more recent review study reported that over 30% of all shunts implanted in adult hydrocephalus patients fail after two years [22].

In the face of the currently suboptimal therapeutic options for hydrocephalus, patients and clinicians have been asking for more sophisticated solutions and have envisioned a “smart shunt” that could provide sensor-based diagnostics, advanced control, and wireless communication [23–26]. As a contribution towards smart shunt systems, this work introduce VIEshunt: a ventricular intelligent electromechanical shunt system. The vision of VIEshunt is a smart shunt system that can monitor relevant physiological parameters, analyze the condition of the patient, drain CSF safely, and adapt to the patient’s actual needs. The aim of this study is to introduce the first prototype of VIEshunt and present the initial results of the development of a smart shunt system for hydrocephalus therapy. We describe the hardware development and controller design of VIEshunt, demonstrate its capabilities using a hardware-in-the-loop (HiL) test bench, and test the system in an in vivo pilot study. Finally, we put this contribution in perspective by providing a concise survey of previous efforts towards the development of a smart shunt.

## Methods

The development of the first VIEshunt prototype was based on the following technical requirements:

1. A smart shunt must be able to measure and control the CSF drainage rate. This includes the prevention of CSF backflow and drainage of CSF against a positive pressure gradient.
2. A smart shunt must be able to measure ICP and regulate it towards pre-defined reference values by adjusting the CSF drainage rate.
3. A smart shunt must be able to adapt to external changes, such as patient posture.

While this list of requirements is certainly not exhaustive, it maintains the basic technical standards of passive shunt systems [27, 28] and captures the main features of an envisioned smart shunt system for hydrocephalus shunt therapy.

### Hardware development

**Fig. 1:**
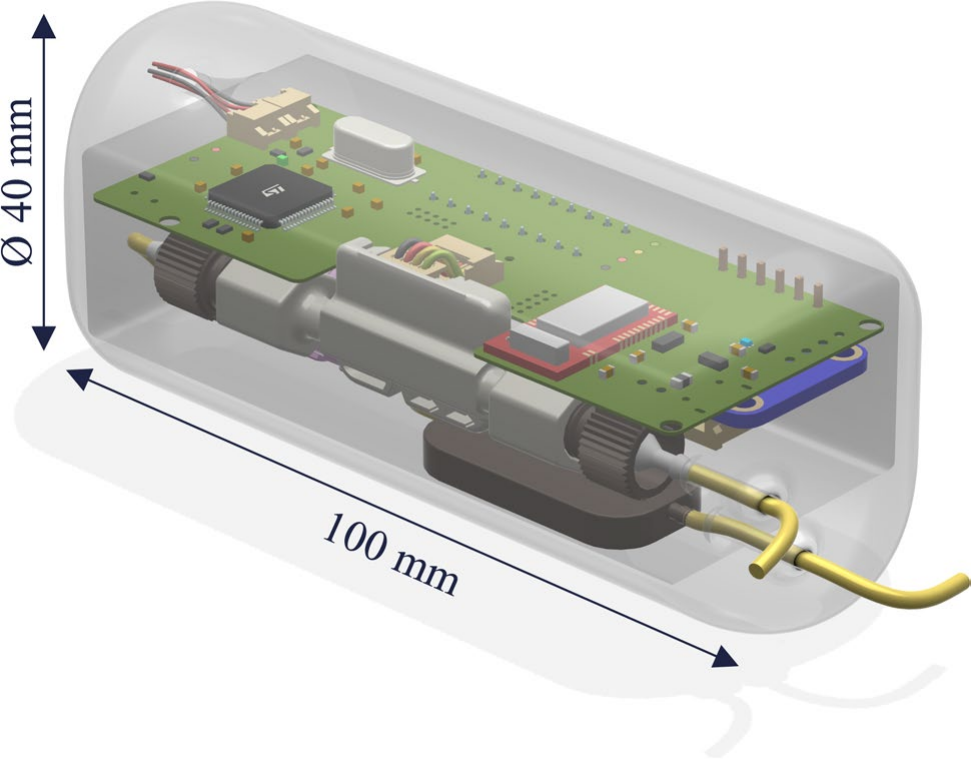
3D rendering with technical components and dimensions of the first VIEshunt prototype

For the development of the first VIEshunt prototype, only commercially available products were used. The individual components were selected to meet the initially defined functional requirements, to ensure safe operation of the overall system, and to achieve the best possible control performance. A piezoelectric micro pump (Bartels Mikrotechnik mp6, Dortmund, Germany) was chosen as the actuator of the shunt system to have full control over the CSF drainage. This pump can generate flow rates up to 8 mL/min at a resolution of around 0.01 mL/min and has a power consumption of around 0.1 W. To ensure that no backflow can occur, the backflow resistance of the micro pump was augmented with a miniature check valve (Smart Products Series 500, Mills River, NC, USA). A flow sensor (Sensirion SLF3S-0600F, Stäfa, Switzerland) that can measure flow rates up to 2 mL/min with 5% accuracy at a 50 Hz sampling rate was integrated to measure the CSF drainage rate for patient monitoring and feedback-based flow control. An optical pressure sensor (Opsens OPP-M200, Québec, Canada) with 1 mmHg accuracy and a 250 Hz sampling rate was used for direct measurement of ICP within the ventricle for improved patient monitoring and feedback-based pressure control. An inertial measurement unit (IMU) chip (Bosch BNO055, Gerlingen, Germany) was integrated to monitor the patient movement and detect posture changes that can influence CSF drainage. To enable wireless communication, a Bluetooth Low Energy (BLE) chip (Nordic Semiconductor nRF51822, Trondheim, Norway) was added. A low-power microcontroller (STMicroelectronics STM32L431RCT6, Geneva, Switzerland) was chosen as the computational unit. All electronic components were integrated by designing a customized printed circuit board (PCB). The PCB, pump and flow sensor were mounted in a custom-made enclosure, which was manufactured via 3D printing using medical-grade silicone with a 60 A shore hardness. A detailed 3D rendering of the first VIEshunt prototype with its components is shown in Fig. 1.

### Controller design

**Fig. 2:**
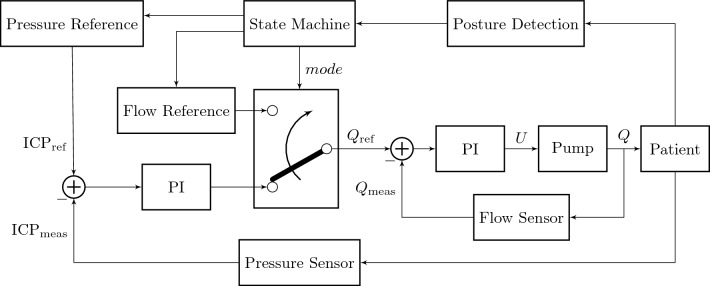
Control architecture for posture-dependent regulation of the patient’s intracranial pressure (ICP) and the shunt’s cerebrospinal fluid (CSF) drainage rate. The state machine uses posture detection information to switch between pressure-based and flow-based control. In pressure-based control mode, the outer control loop uses a proportional-integral (PI) controller to compute a reference drainage rate $Q_{\textrm{ref}}$ based on the difference between the provided ICP reference $\textrm{ICP}_{\textrm{ref}}$ and the ICP measurement $\textrm{ICP}_{\textrm{meas}}$. The inner control loop computes the pump input voltage *U* based on the difference between the drainage reference $Q_{\textrm{ref}}$ and measured drainage $Q_{\textrm{meas}}$. In flow-based control mode, the drainage reference $Q_{\textrm{ref}}$ is directly provided to the inner control loop

Due to VIEshunt’s integrated sensors and active pump, various control strategies can be deployed on the embedded controller. Figure 2 depicts the implemented control architecture for posture-dependent ICP regulation and CSF drainage control. Here, a finite state machine (FSM) is used to switch between pressure-based and flow-based control. In pressure-based control mode, an ICP reference ICP_ref_ that can be pre-defined by the physician is compared to ICP_meas_, the real ICP measured by the pressure sensor. In this outer pressure control loop, a proportional-integral (PI) controller uses the resulting pressure difference to compute a flow reference $Q_{\textrm{ref}}$. As only the regulation of the mean ICP is of interest, i.e., cardiac and respiratory based dynamics of ICP shall not influence the controller action, a 30 s averaging window is applied to ICP_meas_ to filter out waveform effects and other disturbances before the flow reference $Q_{\textrm{ref}}$ is updated. In the inner flow control loop, this flow reference value is compared to the real flow rate through the shunt $Q_{\textrm{meas}}$, measured by the flow sensors. The inner PI controller uses the resulting flow difference to compute and apply *U*, the input voltage of the micro pump, at a rate of 1 Hz. In flow-based control mode, the drainage reference $Q_{\textrm{ref}}$ is directly provided to the inner control loop. In this case, the outer pressure control loop is deactivated and may only serve as a safety feature that interrupts whenever the ICP leaves a pre-defined safety range set by the physician. The PI controller of the outer pressure control loop is mathematically defined as

1 $$\begin{aligned} Q_{\textrm{ref}} = K_{\textrm{p1}} \cdot e_{\textrm{ICP}} + K_{\textrm{i1}} \cdot \int e_{\textrm{ICP}}, \end{aligned}$$

where $K_{\textrm{p1}}$ and $K_{\textrm{i1}}$ represent the controller’s proportional and integral constants, respectively. The current error $e_{\textrm{ICP}} = \textrm{ICP}_{\textrm{ref}} - \textrm{ICP}_{\textrm{meas}}$ is the deviation of the measured ICP from the reference value. Analogously, the PI controller of the inner flow control loop is defined as

2 $$\begin{aligned} U = K_{\textrm{p2}} \cdot e_{Q} + K_{\textrm{i2}} \int e_{Q}, \end{aligned}$$

where $K_{\textrm{p2}}$ and $K_{\textrm{i2}}$ represent the controller’s proportional and integral constants, respectively. The current error $e_{Q} = Q_{\textrm{ref}} - Q_{\textrm{meas}}$ is the deviation of the measured CSF drainage flow from the reference value. In both control loops, an anti-reset-windup is implemented to account for input saturation.

**Fig. 3:**
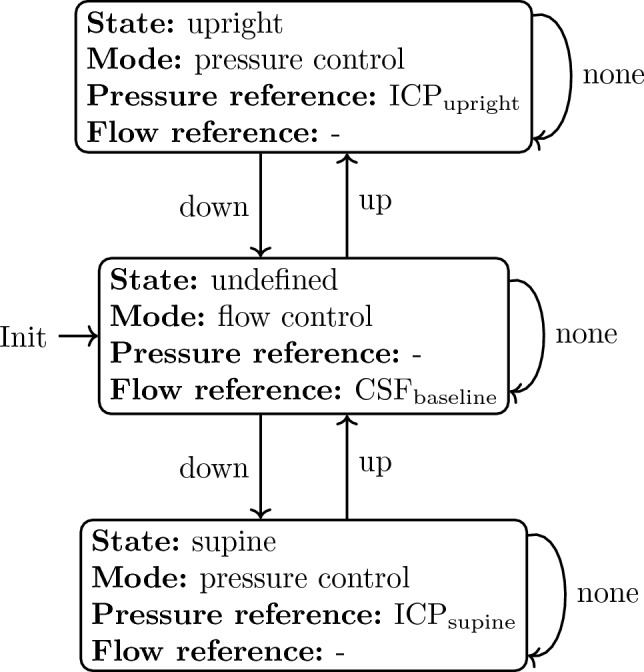
Finite state machine (FSM) for posture-dependent regulation of the patient’s intracranial pressure (ICP) and the shunt’s cerebrospinal fluid (CSF) drainage rate. The patient’s torso inclination is tracked with the shunt’s integrated inertial measurement unit and used to switch between the states upright, undefined, and supine. Depending on the state, the controller mode of the shunt changes and different reference values for the ICP or CSF drainage rate are output

The FSM within this control approach uses the IMU measurements to keep track of the patient’s torso inclination and detects different patient postures, such as the upright, supine, or undefined position. The supine and upright position refer to torso inclination angles of 0 ± 10$^{\circ }$ and 90 ± 10$^{\circ }$, respectively (0$^{\circ }$ represents horizontal). The undefined body position refers to all other torso inclination angles that are outside these two ranges. If the measured torso inclination angles of the patient leave the range that corresponds to the current state upwards or downwards for a predefined time, then the respective up or down input (otherwise none) is applied to the FSM and causes a state transition. Depending on the current state, the state machine’s output provides a reference value for either the ICP or directly for the CSF flow. Assuming a posture-independent overproduction of CSF, the CSF flow reference in the undefined case, e.g., when walking and moving, may be defined as the mean of the logged CSF drainage by the active shunt during times in upright and supine position. The finite state machine is also shown in Fig. 3 and can mathematically be described as $(S, S_0, \Sigma , T, O, G)$, where *S* is the set of states, $S_0$ the initial state, $\Sigma$ the set of inputs, *T* the transition function, *O* the set of outputs, and *G* the output function.

$$\begin{aligned} S&= \{\textrm{upright},\, \textrm{undefined},\, \textrm{supine}\}, \\ S_0&= \textrm{undefined}, \\ \Sigma&= \{\textrm{up},\, \textrm{none},\, \textrm{down}\}, \\ T&= S \times \Sigma \rightarrow S, \\ O_1&= \{\textrm{ICP}_{\textrm{upright}},\, \textrm{CSF}_{\textrm{baseline}},\, \textrm{ICP}_{\textrm{supine}}\}, \\ O_2&= \{\mathrm {pressure \, control},\, \mathrm {flow \, control}\}, \\ G&= S \rightarrow O_1 \times O_2. \end{aligned}$$

### In vitro testing

A previously custom-built HiL smart shunt test bench was used for in vitro shunt testing [13, 29]. The HiL test bench has two pressure-controlled fluid compartments that can simulate the fundamental dynamics of ICP and IAP. These two pressure compartments are furthermore connected by a joint and two beams, the length of which corresponds to the patient’s hip-to-shoulder and shoulder-to-eye distances. While the length of the beams can be adjusted to specific patients, they were chosen to match the dimensions of the average adult American male in this study [30]. Two integrated motors, one connected to the neck joint and the other to the hip-to-shoulder beam, allow for simulation of the rotational movements of the head and the torso. Shunt systems can be mounted at any position along either of the two beams to resemble various implantation locations. In this study, the VIEshunt prototype was mounted on the hip-to-shoulder beam to mimic a placement of the VIEshunt in the abdomen. The fluid that is drained from the ICP into the IAP compartment via the mounted shunt system is measured by a flow probe. Using a return line pump, an equal flow is directed back from the IAP into the ICP compartment such that the test setup allows for continuous long-term testing. During in vitro testing, the VIEshunt prototype was set up for wireless communication with a host computer to transmit measurement data in real time.

A 24 h test was conducted to test the performance of VIEshunt in vitro. During the test scenario, a CSF overproduction was simulated and arbitrary daily posture changes of the patient were mimicked by altering the torso inclination angle between 0–90$^{\circ }$. A numerical patient simulation, which takes the patient posture and flow through the shunt as inputs, was run in real time to compute dynamical pressure changes of the ICP pressure compartment. Depending on the patient posture, different reference values were defined for the shunt controller. An ICP reference pressure of —3 mmHg and 12 mmHg was chosen for upright and supine position, respectively, to reflect pressure values observed in clinical evaluations of postural effects on ICP [9]. For the state with undefined posture, i.e., neither upright nor supine, a CSF drainage reference of 100 $\mu$L/min was defined. Throughout the experiment, IAP was controlled to be 6 mmHg in supine position and 20 mmHg in upright and all other positions [31].

### In vivo testing

Large animal models enable the replication of complex pathophysiology and the testing of medical devices under realistic conditions that facilitate the knowledge transfer to applications in humans [24, 32]. The previously used animal models for hydrocephalus research and in vivo shunt testing are mostly based on kaolin [33–35]. The exact impact on the brain tissue and physiological processes due to the injection of kaolin into the ventricular system is not readily controllable and can result in high morbidity and mortality rates [36–38]. For this reason, the experimental protocol for in vivo shunt testing described in the following was based on a previously introduced ovine model to study the physiologic CSF dynamics [39–43].

#### Ethical statement

Animal housing and all experimental procedures were approved by the local Committee for Experimental Animal Research (Cantonal Veterinary Office Zurich, Switzerland) under the license number ZH135/2020, and were conforming to the European Directive 2010/63/EU of the European Parliament and the Council on the Protection of Animals used for Scientific Purposes, as well as to the Guide for the Care and Use of Laboratory Animals [44]. All health status and housing complied via formal attestation with the Swiss Federal Food Safety and Veterinary Office regulations.

#### Animal preparation

One female white alpine sheep (Ovis gmelini aries) was used for the investigation in this pilot study. The sheep was mature (3 years and 3 month) and had a body weight (BW) of 78 kg. On the day of surgery, the animal was pre-medicated intravenously with 3 mg/kg BW ketamine hydrochloride (Ketasol^®^-100, Dr. E. Graeub AG, Bern, Switzerland) and 0.3 mg/kg BW midazolam (Dormicum^®^, Roche Pharma, Reinach, Switzerland). Prior to orotracheal intubation, anesthesia was induced with 2–5 mg/kg BW of propofol (Propofol^®^-Lipuro 1%, B. Braun Medical AG, Sempach, Switzerland). Anesthesia was maintained throughout the trial by inhalation of 1–2% isoflurane (Attane^®^ Isoflurane ad.us.vet., Piramal Enterpr. India, Lyssach, Switzerland) via volume-controlled ventilation (fresh gas flow 1–1.5 L/min, 18 breaths/min, tidal volume 10–15 mL/kg BW, FiO2 0.7, Pmax 30 mmHg in an oxygen/air mixture) through a semi-closed breathing circuit (Dräger Primus^®^, Dräger Medical, Lübeck, Germany) and balanced with a constant rate infusion of propofol at 2–4 mg/h/kg BW. Intraoperative analgesia was provided by a constant rate infusion of sufentanil (Sufenta^®^ Forte, Janssen-Cilag AG, Zug, Switzerland) at 2.5 $\upmu$g/h/kg BW. Ringer solution (Ringerfundin^®^, B. Braun Medical AG, Sempach, Switzerland) was administered intravenously at an infusion rate of 5 mL/h/kg BW. A small frontal burr hole trephination was performed using a diamond drill in order to access the sheep’s left lateral ventricle and insert the shunt system’s drainage catheter, whereas the shunt system itself remained extracorporeal. A bone wax plug (Ethicon^®^ Bone wax, Johnson & Johnson Medical Ltd., Livingston, United Kingdom) was molded around the exiting catheter to plug the burr hole in the cranium, thus ensuring proper fixation, avoiding leakage of CSF and alterations in ICP. To ensure maximum reliability of the data transfer under demanding laboratory conditions, the in vivo test was performed with a wired connection between the VIEshunt prototype and the host computer.

#### Experimental protocol

In the beginning of the experiment, the VIEshunt was used for ICP monitoring only. During this period, the shunt system’s controller was deactivated and no CSF was drained from the lateral ventricle. Only at the time of about 8 min, the controller was activated and an ICP value of 2 mmHg below the mean ICP value of the initial measurement period was set as a reference for the controller. In order to test the shunt system under pathological conditions, CSF dynamics that are characteristic for hydrocephalus, such as the excessive accumulation of CSF and rise of ICP, were modelled in vivo. To this end, intraventricular bolus infusions with 1 mL of saline solution (NaCl 0.9%) were administered using the implanted drainage catheter. A three-way stopcock was used to switch the connections and prevent infusion of saline solution into the shunt system. The first two bolus infusions were performed at about 21 min and 27 min with the VIEshunt controller activated. At about 36 min, the controller was deactivated, and another bolus infusion was given immediately afterward. Between 45–62 min, the controller was activated for the second period with the same ICP reference value as before.

## Results

### In vitro test results

**Fig. 4:**
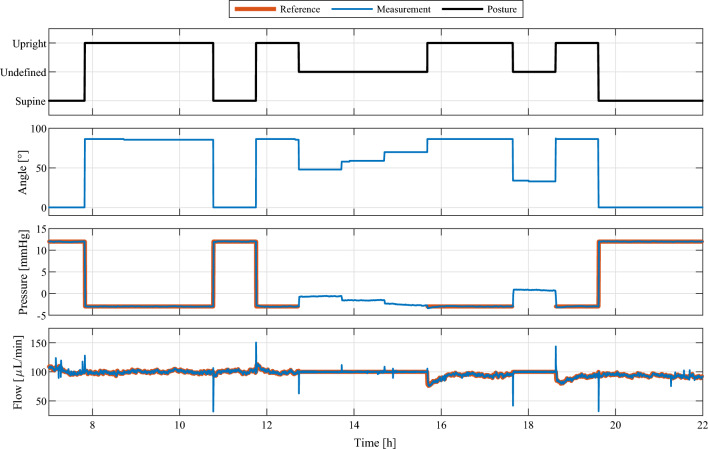
Extract of the in vitro test result. A hardware-in-the-loop test bench with real-time patient simulation was used to simulate changes in the patient’s posture and intracranial pressure for 24 h. VIEshunt automatically switches between the three different posture states (first panel) based on the measured torso inclination of the patient (second panel). The reference values for the intracranial pressure (third panel) and the cerebrospinal fluid flow (fourth panel) are adapted accordingly by the shunt system. In the upright and supine posture state, VIEshunt tracks the pressure reference provided by the state machine and the reference for the low-level flow controller is computed by the high-level pressure controller. In the undefined posture state, the flow reference is directly provided by the state machine and the pressure controller deactivated

**Fig. 5:**
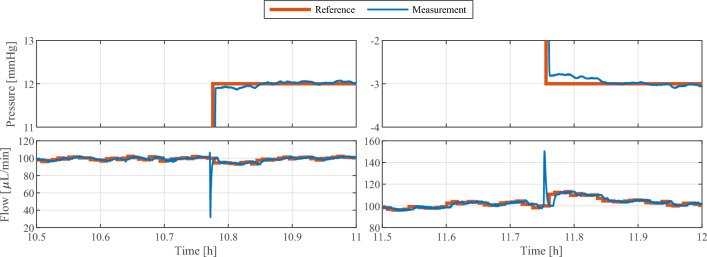
Extract of the in vitro test result, highlighting example posture changes from upright to supine around 10.75 h and supine to upright around 11.75 h. The cerebrospinal fluid (CSF) drainage rate increases for about 30 s during the supine to upright posture change, resulting in an excess outflow of 9.43 $\upmu$L compared to the CSF flow reference. The tracking error of the pressure controller after the posture change ranged between $-$0.1 mmHg and 0.23 mmHg

Figure 4 shows an extract of the 24 h in vitro test of the VIEshunt prototype using the HiL smart shunt test bench. As the 24 h in vitro test aims at mimicking a typical daily activity routine, only the 15 h midday extract with increased activity is shown. The HiL test bench simulated various patient postures by altering the patient’s torso inclination angle between 0$^{\circ }$ and 90$^{\circ }$. The focus was primarily on the supine (0$^{\circ }$) and upright (90$^{\circ }$) position, as well as transitions between those positions. Additionally, intermediate angles such as 33$^{\circ }$, 48$^{\circ }$, 59$^{\circ }$, and 70$^{\circ }$ were also simulated. The inclination angle of the patient’s torso was accurately monitored by the shunt system’s IMU throughout the experiment (see second panel). Using the torso inclination as an input, the FSM detected the posture changes and adjusted its current state (see first panel). Based on the detected patient posture, which was simulated on the test bench, the reference of the shunt controller was automatically adapted. In both upright and supine positions, the ICP could be controlled to the respective reference pressures of —3 mmHg (upright) and 12 mmHg (supine) without undershoots or overshoot in the mean ICP (see third panel). In the undefined posture state, the state machine did not provide a reference pressure to the shunt controller. Instead, the state machine directly provided the reference for the CSF drainage rate to the shunt controller. The shunt controller successfully regulated the CSF drainage rate to the pre-defined reference flow of 100 $\mu$L/min for the undefined posture state of the FSM (see fourth panel). In the supine and upright state, the reference flow rate computed by the outer pressure controller was also tracked successfully and without offset. Changes in the patient’s posture, however, induced short-term disturbances in the CSF drainage rate. Figure 5 shows as an example the change in posture from supine to upright at around 11.75 h. For approximately 30 s, the CSF flow rate was elevated above the controller reference, resulting in an excessive CSF outflow of 9.43 $\upmu$L. During the 15 h extract shown in Fig. 4, the flow controller achieved a root-mean-square error of 1.96 $\upmu$L/min and the integral difference between flow reference and flow measurement averaged out to 3.65 $\upmu$L.

### In vivo test results

**Fig. 6:**
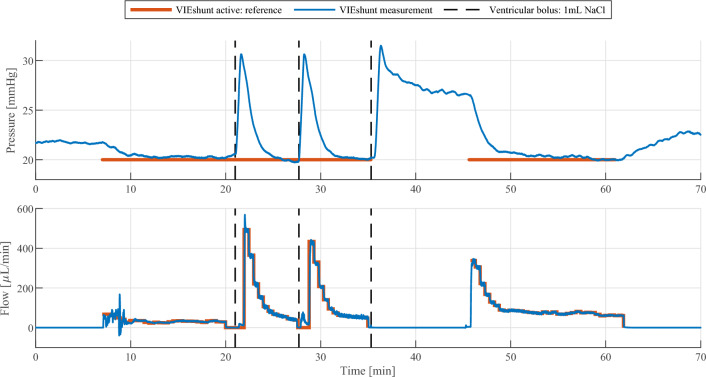
In vivo test results of the acute ovine pilot trial. Intracranial pressure (ICP) and cerebrospinal fluid (CSF) drainage are continuously monitored (shown in blue). VIEshunt is operated in the pressure-based CSF drainage control mode with a 20 mmHg reference value. When the pressure controller is activated (reference plotted in orange), VIEshunt drains CSF and successfully regulates ICP towards the set reference value. During the first two minutes of operation, small flow disturbances are visible due to the initial flushing of the system. At minutes 21, 27 and 36, intraventricular bolus infusions with 1 mL of saline solution are performed (indicated by dashed black lines). Small flow artifacts can be seen around this time as the manually performed interventions used the CSF drainage tubing

The detailed procedure and results of the in vivo pilot trial are shown in Fig. 6. In the initial measurement phase of the trial, i.e., up to 8 min, the ICP fluctuated around 22 mmHg. After the shunt controller was turned on with the pressure reference value set to 20 mmHg, the shunt system started draining CSF and the ICP was successfully regulated to the set reference value. The intraventricular bolus infusions at 21 min and 27 min caused in both cases a pressure increase of about 11 mmHg so that the ICP rose to 31 mmHg. Due to the active shunt drainage, ICP was regulated back to the reference pressure of 20 mmHg in less than 5 min after each of the two bolus infusions. Similar to the two previous bolus infusions, the third bolus infusion also caused a pressure increase of approximately 11 mmHg. In contrast to the previous two bolus infusions, the shunt controller was initially deactivated and only physiological CSF resorption could take place. Although there was a steep decline in ICP in the beginning, the decline slowed down and ICP did not return to the 22 mmHg baseline value of the initial measurement phase when the shunt was also off. In the total period of 9 min following the third bolus infusion, physiological resorption of CSF in fact only led to an ICP decrease of about 5 mmHg. Only once the shunt controller was re-activated at 45 min, the ICP declined below the 22 mmHg baseline value and was again regulated to the controller’s reference pressure of 20 mmHg. When the shunt controller was finally deactivated at 62 min, the ICP rose to about 23 mmHg, which is only slightly above the initial ICP value when the experimental investigation was started.

## Discussion

This work presents the development of the first prototype of VIEshunt: a ventricular intelligent and electromechanical shunt that is capable of posture-dependent CSF drainage control and ICP regulation. The idea for a smart shunt system like ours had already emerged in the 1980s, and a considerable amount of work has been devoted to this research direction ever since. However, most of the previous research and development has focused on individual shunt components or concepts and simulations, rather than implementation and testing. Table 1 provides an overview of the scientific research contributions with a system engineering approach towards the development of intelligent shunt systems for hydrocephalus therapy. Numerous patents also exist in this field, but are not subject to further discussion within this paper. Among the actually engineered prototypes, the shunt systems of Elixmann et al. [45, 46] and Misgeld et al. [34] can be considered the most advanced and integrated. These systems share similar mechatronic features with VIEshunt and are comprised of a microcontroller, an active valve, and sensors for ICP and CSF flow control. However, they were designed to serve as external ventricular drainage systems, which are used in an acute and stationary setting. VIEshunt on the contrary was designed from the beginning with the goal of working towards a medical implant and research platform that allows one to investigate the potential of smart shunts over conventional shunts in chronic in vivo models.

The presented VIEshunt prototype performed successfully in both the 24 h HiL in vitro test and the acute in vivo pilot study. The results show that the technical requirements and functionality for a smart shunt as described in the methods section have been achieved. (1) VIEshunt was able to measure the CSF drainage rate and control it during in vitro and in vivo testing. During in vitro testing, the VIEshunt prototype was able to control the CSF drainage rate at various patient postures (supine to upright) and pressure gradients (positive and negative). (2) VIEshunt was able to measure the ICP and regulate it towards different pre-defined reference pressures during in vitro and in vivo testing. (3) VIEshunt was able to detect changes in patient posture and adapt the controller’s reference value accordingly during in vitro testing. The prototype was also able to react to external disturbances such as bolus infusions of artificial CSF during in vivo testing.

The short-term surges and drops in the CSF flow rate due to posture changes during in vitro testing are assumed to be caused by a shift of liquid within the compliant silicone tubing of the HiL test bench due to the changing gravitational forces when altering the patient posture. While these flow disturbances may appear big in Fig. 4 due to the large timescale, their impact is put into perspective in the example provided in Fig. 5. The integral of the excess CSF drainage during the less than 30 s long peak around 11.75 h is equal to 9.43 $\upmu$L, which is too small to cause an undershoot of the ICP in the underlying real-time patient model of the HiL test bench [13, 29]. The maximum induced CSF flow rate of approximately 150 $\upmu$L/min still remains below the physiological CSF production rate of approximately 300 $\upmu$L/min [47, 48]. The CSF flow disturbances induced by the posture changes of the patient are therefore not considered to have any severe clinical relevance.

As the utilized micro-pump can generate flow rates between zero and up to 8 mL/min, the maximum achievable drainage rate is significantly higher than the natural production rate of CSF at around 0.3 mL/min. Therefore, no technical limitation regarding the achievable flow reference values are expected. Technical limitations regarding the achievable pressure reference values, however, cannot directly be stated as these depend on the CSF dynamics of the specific patient, e.g., the cerebral elastance, CSF production rate, and outflow conductance. In the current VIEshunt prototype design, the definition of the actual controller reference values for the posture-dependent ICP or CSF drainage rate is considered to be at the discretion of the treating physician. How exactly the specific reference values could be determined for each patient individually and how sensory or human feedback could be utilized to adjust the reference values online to adapt to the respective patient over time remain open research questions that are beyond the scope of this study.

**Table 1:** Literature about the conceptualization and development of smart shunts for hydrocephalus therapy

Publications	Contributions
Rekate 1980 [49]	First idea of combining an implant for ICP monitoring with an adjustable valve to achieve closed-loop control of ICP and CSF removal.
Ko et al. 1988 [50]	First concept of a smart shunt system with detailed technical design including valve, telemetry, power supply and sensors for ICP, patient position and CSF flow.
Coté et al. 1995 [51]	Controller design for a potential closed-loop shunt system with an on-off valve and an ICP sensor. Simulation and in vitro system testing using a lumped parameter model of the CSF dynamics and an experimental bench-top apparatus.
Walter et al. 2000 [52], Jetzki et al. 2006 [53]	External ventricular drainage system for ICP regulation with a tube pinch valve, position sensor and connection to commercial ICP monitoring system. Clinical test with five patients up to one week and derivation of an intelligent shunt system concept.
Yoon et al. 2004 [54]	Development and in vitro testing of CSF shunt system with telemetry pressure sensor and MEMS-based micro pump.
Momani et al. 2008, 2009, 2001 [55–57]	Exploration of personalized management systems for CSF drainage and methods for controlling the valve of a smart shunt system. Simulation-based comparison to conventional shunts with a newly derived figure of merit.
Al-Zubi et al. 2009, 2010, 2011 [58–60]	Concept of a management system for hydrocephalus therapy and intelligent shunts with active valves, which can adapt to the acquired biomedical data and human feedback.
Alkharabsheh et al. 2009, 2010, 2013 [61–64]	Concept of a smart shunt system with expert and multi-agent systems using patient feedback for therapy management and shunt diagnosis. Simulation and comparison of potential valve schedule schemes.
Krause et al. 2010, 2011 [65, 66] and Elixmann et al. 2011 [67]	Mechatronic external ventricular drainage system with tube pinch valve and sensors for CSF flow, ICP and bed position. Controller design for ICP regulation, compliance computation and in vitro testing. Extension of [52, 53].
Elixmann et al. 2012 [68]	Comparison of existing shunt valves and future electromechanical designs using CSF dynamics simulations.
Elixmann et al. 2013 [45, 69] and Misgeld et al. 2015 [34]	Robust control approach for an external ventricular drainage system with tube pinch valve and sensors for CSF flow, ICP and bed position. In vivo testing using a porcine model with kaolin-induced hydrocephalus. Extension of [65–67].
Elixmann et al. 2014 [46]	Control of an electromechanical shunt by extracting compliance-related features from the ICP pulse waveform. Implementation and in vitro testing using a shunt prototype with a ball-in-cone valve whose opening pressure can be adjusted using a piezomotor.
Webster et al. 2018 [70]	Concept of a smart shunt system and initial results of the development of a capacitive silicon sensor for ICP and a valve driven by a piezoelectric actuator.
Salih et al. 2019 [71] and Messina et al. 2021 [72]	Concept of a smart shunt system with focus on required hardware. Analysis of the user’s acceptance of healthcare technology based on questionnaire. Design of a valve mechanism and numerical simulation of ICP under shunting.

### Study limitations

The objective of this study was to provide a proof-of-principle for the newly introduced VIEshunt as a smart shunt system for hydrocephalus patients. Within the scope of this study, the VIEshunt prototype was therefore not compared to any commercially available shunt or ASD during in vitro or in vivo testing. For the same reason, the in vivo pilot study was limited to a single animal, in line with the 3R (Replacement, Reduction, Refinement) principles for animal testing [73, 74]. The presented work is not a pre-clinical study and does not provide any statistically relevant conclusions about the potential clinical performance of VIEshunt.

### Next steps towards a smart shunt

The initially defined critical functions and technical requirements of a smart shunt have been met by the first VIEshunt prototype. In the future, several technical developments can be addressed to further improve VIEshunt. These include not only shunt-specific actuator and sensor technology, but also previously unconsidered aspects that are required for the development of a proper medical implant.

*Actuation:* Essentially all previously introduced concepts and prototypes of smart shunts utilize electromechanical valves that can actively alter the CSF flow resistance. The presented VIEshunt prototype uses a piezoelectric micro pump that in combination with a flow sensor enables accurate and precise drainage of CSF, regardless of the pressure gradient acting along the shunt. In general, micro pumps are becoming increasingly common in biomedical applications and various types exist that differ in terms of driving voltage, power consumption, operating frequencies, flow rate, back pressure and size [75]. The employed micro pump in this study was not explicitly designed for CSF drainage control, however, research and development for dedicated micro pumps is an active field and could mitigate problems like high power consumption and operating voltages [76].

*Flow:* The presented prototype uses one of the few commercially available mass flow sensors capable of resolving the low flow rates of the CSF drainage. Flow sensors specialized for the application in hydrocephalus therapy have been investigated for several decades, and further developments can be leveraged to improve the measurement quality, reduce the sensor size or integrate additional functionalities such as detection of catheter clogging [77–91].

*Pressure:* ICP measurement is not only a central component of hydrocephalus diagnosis but also of patient monitoring and has led to the development of a variety of telemetric systems for long-term ICP monitoring [92]. However, ICP sensors suffer from technical limitations that become even more critical when used in an implantable device, e.g., sensor drift, reference pressure variability, and signal quality control [93]. While the chosen fiber optic measurement system offers high accuracy plus reduced sensor drift and avoids the pressure disturbances experienced by pressure sensors that are placed in-line with the drainage flow, it requires an additional bulky transducer module. The development of improved ICP measurement systems is an active research field, and new sensors could be integrated in future prototype iterations [94, 95].

*Bioimpedance:* Normal pressure hydrocephalus is a type of hydrocephalus in which the mean ICP remains within the physiological range despite excessive accumulation of CSF and ventricular enlargement. In this case, ICP measurements alone may not be sufficient for patient monitoring and therapy management. To address this issue, bioimpedance measurements that exploit the difference in electrical conductivity of CSF and brain parenchyma in order to monitor ventricular volume changes have been proposed and could be incorporated in future prototypes as an additional measurement modality [96–100].

*MRI:* Since magnetic resonance imaging (MRI) is an essential method to examine the health condition of patients in various diseases, MRI safety is an important factor in the development of medical implants [101]. While MRI conditional shunts and electronic implants do exist, this was not a focus of the current work and will need to be addressed in the development of future VIEshunt prototypes.

*Power:* The presented prototype still relies on an external power supply, but future prototypes will need a battery-based power supply with wireless energy transfer to allow for chronic system testing [102–104].

*Size:* Contemporary shunt systems are sufficiently small to allow for the direct implantation in the skull. Due to their mechatronic nature, future smart shunts will certainly be larger and need to be implanted in different locations, such as the chest or abdomen. While the size of the current VIEshunt prototype is too large for application in humans, it is however feasible for the use in chronic large animal studies. In future prototypes, a compact design and system integration will be of higher priority in order to reduce the implant’s size and weight.

### Remaining smart shunt limitations

With the outlined technical improvements, VIEshunt has the potential to improve hydrocephalus therapy by ensuring physiological CSF drainage, enabling continuous patient monitoring, and reducing shunt malfunction. However, it is important to note that two major sources of shunt failure are not fully addressed and may remain even then: obstruction and infection. In future smart shunt systems, this might be mitigated by detecting catheter obstructions and infections using smart sensors [64, 85, 105–109]. While the shunt can make an invaluable contribution by communicating these complications to the patient or physician, it will not necessarily be able to directly remedy them. The best approach should be to avoid shunt complications in the first place by prioritizing a holistic shunt development approach that further improves catheter design and infection preventing mechanism [110]. Catheter design can, e.g., be improved by using computational fluid dynamics [111–113] and integration of clearing mechanism [114, 115]. On the other hand, shunt infections can be reduced by using antimicrobial shunt catheters [116] and following dedicated infection prevention protocols [117, 118]. Importantly, any smart shunt must always be seen as only one element of an overarching clinical patient management approach [119].

## Conclusion

Since their clinical introduction, the design and functionality of shunt systems for hydrocephalus therapy have changed only in incremental steps. Smart shunt systems for hydrocephalus therapy have been envisioned and demanded for several decades already, but despite the tremendous technological advances made in this time, such a system does not yet exist on the market. Most of the research in this direction is focused on concepts and individual components, rather than holistic approaches and prototype development. To advance the progress towards a smart shunt system for hydrocephalus therapy, this study presents the development and testing of the first prototype of VIEshunt: a ventricular intelligent and electromechanical shunt system. This includes the combination of an active pump with an embedded microcontroller that uses sensory feedback of ICP, CSF drainage and patient posture to facilitate posture-dependent control of CSF drainage and ICP regulation. A proof-of-principle for this VIEshunt prototype has been accomplished in vitro using a HiL test bench and in vivo using a large animal model. Future shunts based on VIEshunt could improve patient monitoring and enable optimal physiologic therapy by adapting to measured parameters of the patient. However, several development and validation tasks remain that need to be addressed before VIEshunt emerges as a true smart shunt system that could be used clinically to improve therapy for hydrocephalus patients. Future work will focus on integrating a battery-based power supply, improving the embedding of all mechatronic components, extending the wireless communication capabilities to incorporate user feedback, and developing advanced algorithms for patient monitoring and shunt system control. Finally, pre-clinical studies with an appropriate sample size and a control group of conventional shunts will need to be conducted to validate VIEshunt and to derive statistically relevant conclusions about its performance.

## Data Availability

The data of this study are available from the authors upon reasonable request.

## Code availability

The code of this study is available from the authors upon reasonable request.
